# The Association Between Daily Registrations of Persistent Post-Concussion Symptoms Using an mHealth App and the Retrospective Rivermead Post-Concussion Symptoms Questionnaire

**DOI:** 10.1177/2689288X251372548

**Published:** 2025-09-04

**Authors:** Johanne Rauwenhoff, Gøril Storvig, Bert Lenaert, Anker Stubberud, Toril Skandsen, Erling Tronvik, Alexander Olsen

**Affiliations:** ^1^ Department of Psychology, NTNU Norwegian University of Science and Technology, Trondheim, Norway.; ^2^ NorHead, Norwegian Center for Headache Research, Trondheim, Norway.; ^3^ Clinic of Rehabilitation, St. Olavs Hospital, Trondheim, Norway.; ^4^ Department of Lifespan Psychology, Open University, Heerlen, The Netherlands.; ^5^ Department of Health Promotion, Faculty of Health, Medicine and Life Sciences, Maastricht University, Maastricht, The Netherlands.; ^6^ Department of Neuromedicine and Movement Science, NTNU Norwegian University of Science and Technology, Trondheim, Norway.; ^7^ Neuroclinic, St. Olavs Hospital, Trondheim, Norway.

**Keywords:** daily and retrospective registrations, mHealth, mild traumatic brain injury, persistent post-concussion symptoms

## Abstract

A substantial number of people experience persistent post-concussion symptoms (PPCS) following a concussion. Traditional retrospective assessments, such as the Rivermead Post Concussion Symptoms Questionnaire (RPQ), are prone to memory biases and do not capture the day-to-day variability of PPCS. In this study, we explored the association between daily registrations of PPCS and the RPQ. We also examined the variability of PPCS trajectories over time. Nineteen participants registered PPCS symptoms for 28 days using an mHealth app and then completed the RPQ. From the final 7 days, average, highest, and last-day symptom scores were calculated and correlated with corresponding RPQ items. Scores from the full 28-day period were used to compute the within-person standard deviation and mean squared successive difference (MSSD) for each symptom that participants rated as the most bothersome. Correlations between the RPQ and daily registrations were weak-to-medium (range: 0.343, 0.590). The retrospective RPQ explained up to 35% of the variance in average daily registrations of PPCS. The MSSD ranged from 0 to 16.29, and the within-person SD from 0 to 3.25. Visual analyses showed that participants with identical RPQ item scores often exhibited different PPCS variability. This was also true for different symptoms within the same participant. This study highlights the potential additional value of daily registrations for capturing the dynamic and fluctuating nature of PPCS, which may be missed by retrospective questionnaires administered at one time point. PPCS vary both within and between individuals over time and reducing this complexity to a single total score oversimplifies a nuanced reality. Larger studies are needed to confirm these findings, and future work should investigate the clinical relevance of capturing daily variations in PPCS.

## Introduction

Most people recover spontaneously in the first days or weeks following a concussion. However, there is a subgroup of people who experience persistent post-concussion symptoms (PPCS) months or years after the injury.^1–3^ These symptoms may be somatic, behavioral, or cognitive in nature, including headache, fatigue, and memory problems.^4^ PPCS have a negative impact on daily functioning, may cause distress, and reduce quality of life.^5^ The etiology of PPCS is not fully understood but may be explained through a biopsychosocial framework.^6^ This framework highlights the possible interaction of various pre-injury vulnerabilities (such as sex), acute physiological responses (such as inflammation), and post-injury influences (such as coping mechanisms).^7–9^ The experience of PPCS can differ significantly from individual to individual and may fluctuate daily, making assessment, classification, and management challenging.

Enhanced understanding of PPCS is crucial for improving diagnostics and treatments. This effort is limited by current methods used to assess PPCS, which typically depend on retrospective memory self-reports. When completing retrospective questionnaires, people need to rely on their memory, which is prone to several forms of memory bias.^10^ People often recall past experiences in ways that support future decisions, prioritizing adaptive interpretations rather than ensuring accuracy.^10,11^ Retrospective reports might be influenced by the thoughts and beliefs a person has about their health. For instance, the Rivermead Post-Concussion Symptoms Questionnaire (RPQ),^12^ a retrospective questionnaire often used to measure PPCS, asks individuals to compare their current state with their pre-injury functioning. While one could question whether people truly consider the comparison to pre-injury functioning when completing the questionnaire, this reference point can still contribute to overreporting due to the “good-old-days bias,” the tendency to recall one’s pre-injury state as better than it actually was.^13^ In addition, the peak-end effect leads individuals to recall the most intense and recent experiences better, while the memory-experience gap states that symptoms are often overreported when assessed retrospectively.^11^ Moreover, people with PPCS can experience cognitive problems, which can also influence retrospective reports. Lastly, questionnaires administered once do not capture the day-to-day fluctuations in symptoms, likely offering an incomplete picture of the PPCS experience. However, daily variability in PPCS has not yet been thoroughly investigated in individuals with PPCS.

It is thought that in-the-moment registrations are more closely related to objective physiological measures of biobehavioral processes, as these are based on here-and-now information without having to rely on memory.^10^ In a clinical setting, daily symptom tracking could help capture the day-to-day fluctuations of symptoms, assist in the identification of symptom triggers, and aid more personalized and timely treatment adjustments. In research, daily tracking allows for a more detailed analysis of symptom and individual variability over time.^14^ Retrospective and daily measurement approaches often yield different results. A study by Kikuchi et al.^15^ found poor consistency between momentary reports and retrospective assessments of headache intensity, especially in individuals with highly variable headache patterns. Headache is one of the most frequently reported PPCS, along with fatigue.^6^ Patients with chronic fatigue tend to recall higher levels of fatigue than their real-time momentary ratings suggest.^16^

We developed and evaluated the feasibility of an mHealth application that allows individuals to register PPCS daily. In the current study, our first aim was to assess the strength of the relationship between the real-time experience of PPCS as measured with the new mHealth application and the commonly used retrospective RPQ.^12^ Our second aim was to examine the characteristics, specifically the variation, of PPCS trajectories over time.

## Methods

### Design

**FIG. 1.**
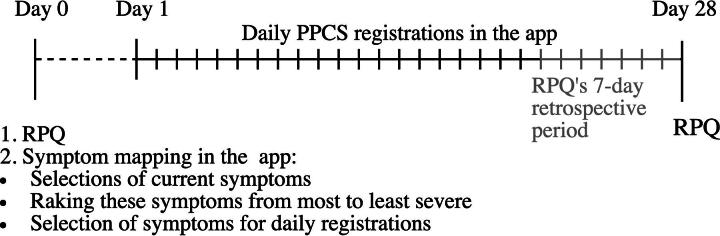
Design of the study.

The study employed a within-subject, observational daily diary design using a personalized mHealth app, with a retrospective self-report measure (RPQ) included for comparison. Participants used an mHealth application for symptom tracking for 28 days (daily symptom registrations) in their home environment. Before this period, participants conducted a “symptom mapping” procedure in the app, consisting of selecting their current symptoms, ranking these symptoms, and selecting symptoms for the daily registrations. As the app is developed for clinical use, participants could choose which symptoms they wanted to track on a daily basis, with the intention to personalize the experience and reduce practical burden. At the end of the study period, participants completed the RPQ, which asked participants to report symptoms experienced over the past 7 days. This created a 7-day overlap between the daily symptom registrations and the retrospective RPQ assessment. The design of the study can be seen in Figure 1.

### Participants

We aimed to include at least 20 participants with PPCS. The sample size was determined based on previous research and recommendations for mHealth studies of usability,^17,18^ which was the primary objective of the original study (ClinicalTrials ID: NCT05635656). Participants were recruited from the outpatient rehabilitation clinic at St. Olavs University Hospital in Trondheim, Norway, from December 25, 2022, to March 5, 2024, through announcements on the hospital’s official website, the Norwegian Center for Headache Research (NorHead) website, and user organizations and their social media. Inclusion criteria were age 18 years or older, mild traumatic brain injury (mTBI) as defined by having sustained a head injury with observed or self-reported alteration of consciousness, amnesia, or other relevant acute symptoms, PPCS according to the International Classification of Diseases (ICD-10) research criteria (operationalized as reporting three or more symptoms [including headache] on a moderate or greater intensity level on the RPQ), post-traumatic headache according to International Classification of Headache Disorders 3rd edition,^19^ proficiency in Norwegian language, and signed written informed consent. Exclusion criteria were severe psychiatric or somatic disease or other patient-related factors that would provide obvious challenges for adhering to the study protocol (including using the mHealth solution), having no access to an iOS or Android smartphone, or having less than 3 months of experience with smartphones. Signed written informed consent was obtained from all study participants. The study was approved by the Norwegian Medicinal Products Agency (MDP 22/16250-7) and the Norwegian Ethics Committees for Clinical Trials on Medicinal Products and Medical Devices (REC-KULMU 422538), and pre-registered in ClinicalTrials.

### Outcomes

#### Symptom mapping and daily registration of PPCS

**FIG. 2.**
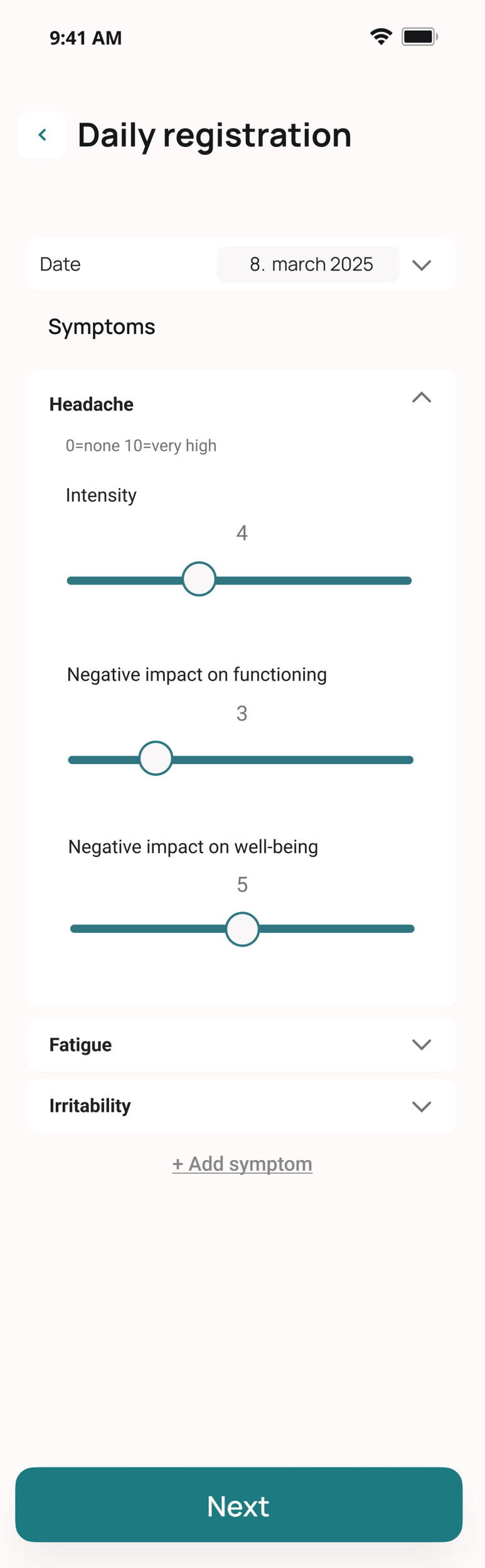
Screenshot of the mHealth application in which participants registered their PPCS. The drop-down menu for each symptom contains three different parameters: intensity of symptoms, impact on functioning, and impact on well-being. PPCS, persistent post-concussion symptoms.

For daily symptom registration, we used an in-house developed mHealth application, owned by St. Olavs Hospital and NTNU. St. Olavs Hospital and NTNU contracted Nordic Brain Tech AS (Oslo, Norway) and Fominykh Consulting (Trondheim, Norway) for consultant services on app programming. The app demonstrated good usability, feasibility, and safety; for more details, see Storvig et al.^20^ In this study, we used participants’ daily symptom registrations and a onetime symptom mapping procedure. The symptom mapping involved three steps: (1) participants selected the symptoms they currently experienced, (2) they ranked these symptoms from most to least bothersome, and (3) they chose which symptoms to track daily. A full list of symptoms available for tracking is provided in Supplementary Table S1. Using the same app, participants were asked to report their PPCS symptoms daily for 28 days at home. For each selected symptom, they completed three ratings on an 11-point Likert scale (0 = none to 10 = very high): “Intensity of the symptom,” “Negative impact on your daily functioning,” and “Negative impact on your well-being,” measuring intensity of the symptoms, effect on functioning, and effect on quality of life (QoL). Figure 2 shows a screenshot of the symptom registration interface in the app.

#### Rivermead Post-Concussion Symptoms Questionnaire

The RPQ was used to retrospectively assess PPCS by asking participants to rate their physical, cognitive, and behavioral post-concussion symptoms compared with their pre-injury symptoms.^12^ The RPQ consists of 16 items, each rated on a 5-point Likert scale, ranging from 0, “not experienced at all,” to 4, “a severe problem.” Higher scores indicate more symptom experience. As per the original scoring method, when calculating the total score, responses of 1 (no more of a problem than before) were recoded to 0.^12^ The Norwegian version used, approved by the CENTER-TBI Consortium,^21,22^ has shown good psychometric properties^21^ and differs from the original RPQ in using a 7-day rather than a 24-h recall period. The RPQ was chosen for this study because it is one of the most widely used instruments for assessing PPCS in both research and clinical practice.^23^ The RPQ has undergone translation and validation in multiple languages and has been used in large-scale, prospective cohort studies such as the CENTER-TBI in Europe and Transforming Research and Clinical Knowledge in Traumatic Brain Injury (TRACK TBI) in the United States.^24–26^ This makes it a valuable reference point for comparison with the daily symptom registrations. Furthermore, it is recommended for the assessment of PPCS in people with mTBI by the Common Data Elements guidelines of the National Institutes of Health.^27^

#### Demographic and injury information

Demographic and injury-related information was gathered through a telephone interview. The demographic information included sex, age, and education. The patients furthermore provided clinical information on injury-related factors: cause of concussion, time since the injury, duration of loss of consciousness, and duration of post-traumatic amnesia.

### Procedure

Data from the Digital solutions for concussion (DiSCo) study were used (ClinicalTrials ID: NCT05635656). In the DiSCo study, two mHealth solutions for individuals with PPCS were developed, focusing on the safety, usability, and feasibility of the applications. Data from one of the applications, a biofeedback app, is not used in this study, while the mHealth app for self-monitoring PPCS symptoms is used. The DiSCo study consisted of three iterative cycles to develop and refine the apps; the first two cycles were hour-long sessions at the hospital, and the last cycle was a home-testing period of 28 days. The symptom registration data from the 28-day home-testing period were used in the current study.

Symptom mapping was performed first in the app. Hereafter, for 28 days, the participants could register their selected PPCS at any time of the day. They received a notification on their phone with the question “Did you experience symptoms today?” at 8 pm as a reminder. If participants clicked “yes,” they were directed to a screen where they could track their selected symptoms (see Fig. 2). Participants were able to change which symptoms they rated (i.e., removing or adding symptoms) during the study period, with the intention to personalize the experience and reduce practical burden. Furthermore, participants were able to register symptoms experienced within the last 48 h if they did not track those symptoms on those specific days. Study personnel made three scheduled phone calls to assess any technical issues with the app. Additional phone calls were scheduled for further troubleshooting if technical issues were not solved immediately. RPQ was completed on day 0 and day 28.

### Statistical analyses

In order to assess the strength of the association between daily registrations and the RPQ, we compared the RPQ item scores from day 28 with the daily registrations from the preceding 7 days (Fig. 1). From the daily PPCS registrations in the app, we calculated for each registered symptom (1) the average score of the 7 days (“average PPCS”), (2) the highest recorded PPCS score over the last 7 days (“maximum PPCS”), and (3) the PPCS score on the final day (“PPCS last-day”). These indices were calculated for the PPCS symptoms that the participants chose to self-monitor and were subsequently entered into the correlational analyses with the corresponding item on the RPQ (matched RPQ-item score). For instance, if a participant self-monitored fatigue, the indices of average, maximum, and last-day fatigue were compared with the fatigue item on the RPQ. This was done separately for the intensity, QoL, and functioning scores. As the data were not normally distributed, Spearman correlations were used. Bootstrapped 95% confidence intervals were reported.

To investigate the characteristics of the PPCS trajectories further, the worst-ranked symptom (number one symptom) was examined for the whole period of 28 days. The within-person standard deviation and mean squared successive difference (MSSD) were calculated as an index of within-individual change.^28^ These measures were also calculated for the symptoms that were tracked by 10 participants or more. These analyses were performed with only the intensity scores. Furthermore, individual visual analyses were performed to look at the differences in daily registrations for participants with similar RPQ scores.

### Handling technical issues and missing data

The symptom diary app encountered several technical issues during the DiSCo study. In some instances, participants were able to make multiple registrations for the same day (25 registrations, 0.6%). When two or more registrations with different scores occurred on the same day, the highest score was used (which in all cases was also the latest registration for that day). If the registrations for the same day were registered on different days, the one closest in time to the symptom day was selected. Furthermore, due to technical issues, some participants were able to retrospectively track data beyond 2 days. These entries were excluded from the current analyses (181 registrations, 7.3%). Lastly, it was not possible to differentiate between instances where participants entered a score of zero for intensity, functioning, and QoL items, and cases where this data was missing. Cases where participants reported a score of zero for intensity, functioning, and QoL on all symptoms for an entire day (165 registrations, 6.7%), or where a symptom was consistently scored as zero throughout the study period, were therefore excluded from the analysis (62 registrations, 2.5%).

## Results

### Participants

A total of 23 participants were included in the study. Two participants dropped out from the study before starting the daily symptom tracking period, citing personal reasons not related to the study as the reason for dropout. Two other participants encountered technical difficulties, resulting in substantial missing data, and were therefore excluded from the analyses. Consequently, the final analyses included 19 participants. The demographic and injury-related variables can be found in Table 1.

**Table 1.** Demographic and Injury-Related Variables

Demographic Variables	Mean (SD) or *n* (%)
Sex, *n* women (%)	14 (73.7)
Age, mean (SD)	48.3 (13.7)
Education, *n* (%)	
Upper secondary, vocational	4 (21.1)
Upper secondary, general	2 (10.5)
Tertiary education	13 (68.4)

Injury-related variables	Mean (SD) or *n* (%)
Time (years) since injury, mean (SD)	5.3 (4.0)
Injury mechanism, *n* (%)	
Struck by/on object	11 (57.9)
Traffic accident	4 (21.1)
Fall	3 (21.7)
Violence	1 (5.3)
PTA, *n* (%)	
No	2 (10.5)
Less than 1 h	6 (31.6)
Between 1 and 24 h	7 (36.8)
Unknown	4 (21.1)
LOC, mean (SD)	
No	5 (26.3)
Less than 5 min	8 (42.1)
Yes, duration unknown	2 (10.5)
Unknown	4 (21.1)
History of previous concussions, yes (%)	9 (47.4)
Total score RPQ, mean (SD)	35.2 (6.1)
Subscore RPQ somatic	19.1 (3.2)
Subscore RPQ emotional	8.0 (3.8)
Subscore RPQ cognitive	8.1 (2.3)

LOC, loss of consciousness; PTA, post-traumatic amnesia; RPQ, Rivermead Post-Concussion Symptoms Questionnaire; SD, standard deviation.

#### Comparison between RPQ scores and daily registrations of PPCS

There were 16 participants whose PPCS registrations overlapped with the RPQ’s 7-day retrospective period, leading to a total of 72 symptom registration periods for intensity, 68 for daily functioning, and 67 for QoL with an 89.9% compliance rate. Participants contributed varying amounts of data, as they were free to choose how many symptoms to track. An overview of the number of tracked 7-day retrospective periods per person and symptom can be found in Supplementary Tables S2, S3, and S4 for the intensity, functioning, and QoL scores. Headache was the most frequently tracked symptom (tracked by 14 participants), followed by fatigue (10 participants), and concentration difficulties (9 participants). All RPQ items participants chose to track had scores ranging from 2 to 4; no symptoms with an RPQ item score of 0 or 1 were selected for tracking. From the seven-day retrospective period, we calculated the three indices. The mean, range, and SD of these indices and the matched RPQ item scores can be seen in Table 2.

**Table 2.** Mean, Range, and SD of the Intensity, Daily Functioning and Quality-of-Life Indices and the Matched RPQ Item Scores

Index	Indices	Mean	Range	SD
Intensity	Average PPCS	2.20	0–5.67	1.53
(11-point scale)	Highest PPCS	4.74	0–9	2.36
	PPCS last-day	2.57	0–7	2.34
Daily functioning	Average PPCS	1.97	0–5.57	1.49
(11-point scale)	Highest PPCS	4.55	0–10	2.50
	PPCS last-day	2.60	0–7	2.40
Quality of life	Average PPCS	1.71	0–5.71	1.48
(11-point scale)	Highest PPCS	3.94	0–10	2.65
	PPCS last-day	2.29	0–8	2.46
Matched RPQ-item score	—	2.86	2–4	0.64
(4-point scale)				

PPCS, persistent post-concussion symptoms registrations; RPQ, Rivermead Post-Concussion Symptoms Questionnaire; SD, standard deviation.

Table 3 shows the results of the correlation analyses. Results showed weak-to-moderate correlations (range: 0.399–0.590) regarding intensity, weak-to-moderate correlations (range: 0.343–0.522) regarding the impact on daily functioning, and weak-to-moderate correlations (range: 0.353–0.524) regarding the impact on QoL. The retrospective RPQ items explained 35% of the variance in average daily registrations of PPCS for intensity, 27% for daily functioning, and 27% for QoL.

**Table 3.** Correlation Analysis Between the Matched RPQ-Item Scores and the Different Incidences of Daily PPCS Registrations

	Average PPCS	Highest PPCS	PPCS last-day
**Intensity**			
RPQ-item score			
ρ	0.590	0.399	0.429
CI	(0.425, 0.729)	(0.197, 0.593)	(0.205, 0.615)
p	0.001	0.001	0.001
N	72	72	63
**Daily functioning**			
RPQ-item score			
ρ	0.522	0.343	0.361
CI	(0.324, 0.677)	(0.114, 0.540)	(0.093, 0.600)
p	0.001	0.002	0.005
N	68	68	60
**Quality of life**			
RPQ-item score			
ρ	0.524	0.353	0.412
CI	(0.317, 0.695)	(0.122, 0.575)	(0.196, 0.599)
p	0.001	0.003	0.001
N	67	67	59

CI, 95% bootstrapped confidence intervals; PPCS, persistent post-concussion symptoms registrations; RPQ, Rivermead Post-Concussion Symptoms Questionnaire.

#### Characteristics of the trajectories of PPCS

**FIG. 3.**
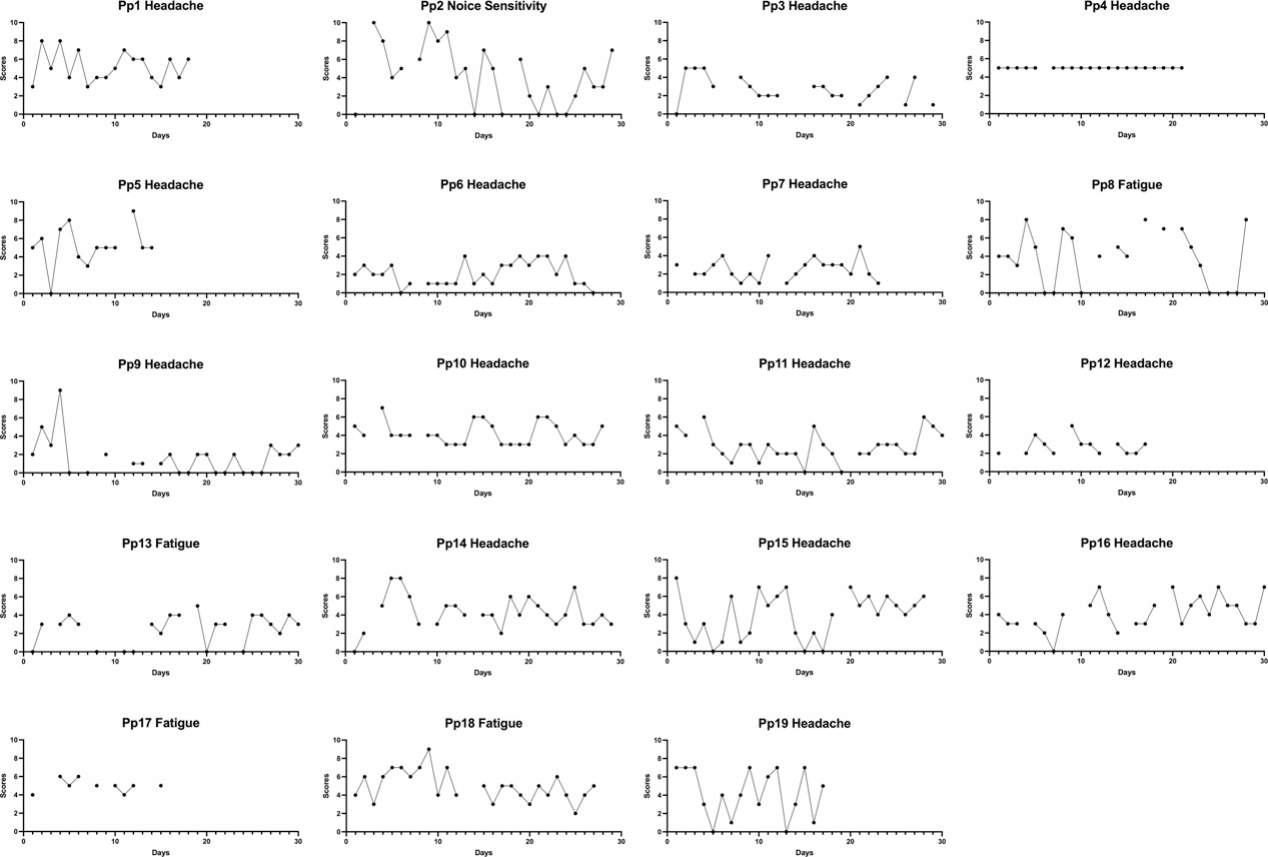
Intensity scores of the most bothersome symptom per participant over 28 days.

Of the 19 participants, 14 indicated that headache was their number one symptom. For four others, this was fatigue, and for one participant, this was noise sensitivity. The total number of registrations per participant varied from 9 to 29. There were two symptoms that were tracked by more than 10 participants, namely headache and fatigue. The range, mean within-person SD, and MSSD can be found in Table 4. The number one or most bothersome symptom per participant is depicted in Figure 3.

**Table 4.** Mean, Mean Squared Successive Difference, and Within-Person Standard Deviation of the Daily Registered Intensity Scores

	*N*	Range	Mean
Number one symptom			
MSSD full period	19	0–16.29	5.73
SD full period	19	0–3.25	1.70
Headache			
MSSD full period	17	0–18.05	6.02
SD full period	17	0–3.10	1.68
Fatigue			
MSSD full period	12	1–22.64	9.60
SD full period	12	0.71–3.01	2.189

MSDD, mean squared successive difference; SD, within-person standard deviation.

**FIG. 4.**
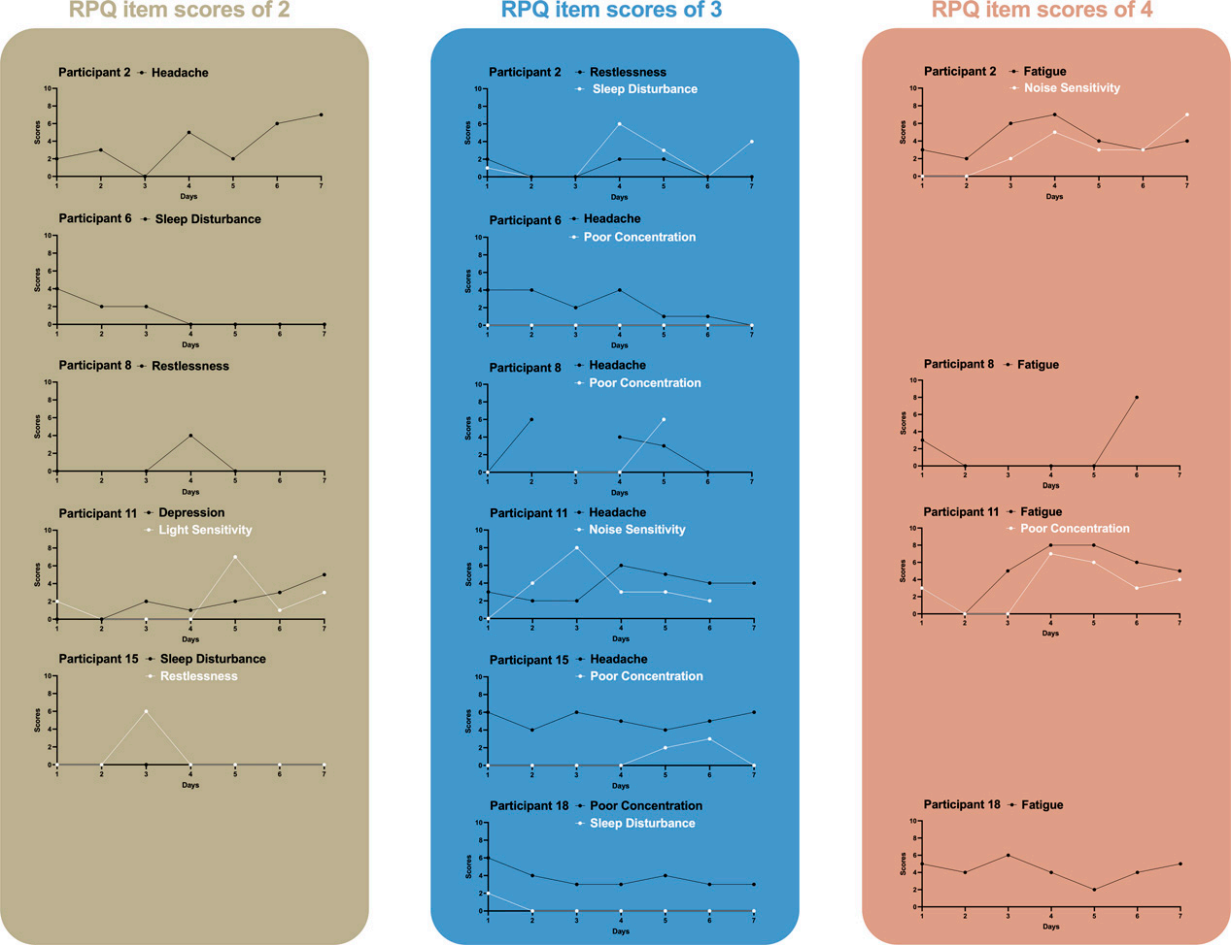
Daily symptom intensity scores presented per participant and RPQ item score. Note for Figure 4: In this figure, the daily PPCS intensity scores of the 7-day retrospective period of the RPQ are shown for six participants, grouped by the RPQ item score for that symptom (as reported on the last day). In order to illustrate the difference in variability, a similar item score on the RPQ can be related to very different daily registrations both between and within the participants. PPCS, persistent post-concussion symptoms; RPQ, Rivermead Post-Concussion Symptoms Questionnaire.

We conducted visual analyses to look at the patterns in PPCS of people with similar RPQ item scores. To illustrate this, Figure 4 shows the daily PPCS registrations for six participants in the RPQ retrospective period (seven-day period), grouped by their RPQ item score. In this figure, we can see that a similar item score on the RPQ can be related to very different daily registrations. For instance, participants 6, 8, 11, and 15 all scored their headache as three on the RPQ, the averages of their daily registrations; however, range from 2.29 to 5. Additionally, there are differences in the amount of variability; for instance, the headache scores of participant 15 range from 4 to 6, while for participant 8, these range from 0 to 6. Furthermore, there are also within-person differences. Participant 15 gave a score of 3 on the RPQ for both headache and poor concentration. However, their average headache score is 5, while the average of their scores for poor concentration is 0.71. Similarly, participant 11 gave a score of 3 on the RPQ for both headache and noise sensitivity. While their headache scores ranged from 2 to 6, their noise sensitivity scores ranged from 0 to 8. Furthermore, when comparing the daily registrations of PPCS in the app with scores of 4 on the RPQ, we observe notable variation. For example, participants 2 and 18 never registered daily symptom scores higher than 6 or 7. Participant 8 initially registered scores of 0 and 3 but recorded an 8 on the final day. Participant 11 not only registered high scores regarding poor concentration and fatigue but also had 0 scores for both symptoms.

## Discussion

In this study, we aimed to compare daily registrations of PPCS using an mHealth application with a widely used retrospective questionnaire, the RPQ. We found weak-to-moderate correlations between the two different measures for PPCS assessment. The RPQ explained up to 35% of the variance in average daily registrations of PPCS. These findings suggest that a onetime retrospective measure, such as the RPQ, may not fully reflect the day-to-day experience of PPCS.

These results correspond to theoretical approaches that explain why retrospective and prospective in-the-moment reporting may differ.^10^ That is, retrospective reports may be influenced by biases such as the peak-end effect or other memory biases. Furthermore, retrospective reports can be influenced by the participants’ thoughts and beliefs about themselves, their health, and their symptoms.^10^ The results of the current study correspond with research in other patient populations, finding weak associations between daily registrations and retrospective measures of individual PPCS^15,16^ and with the study by Lenaert et al.,^29^ who found similarly weak-to-moderate associations between daily and retrospective registrations of fatigue in people with stroke. However, the study of d’Offay et al.^30^ has found a strong correlation (*r* = 0.86) between average post-concussion symptoms as measured with the RPQ and a symptom diary that participants completed once daily for 5 weeks. The study included people directly after they experienced a concussion, which differs from our study, which includes people with PPCS who were, on average, 5 years post-injury. In an early stage after the concussion, it is likely easier for people to compare their symptoms to pre-injury. Additionally, people who have experienced PPCS for a long time can develop negative thoughts and beliefs about their symptoms and, as a result, may have a different illness perception.^9,31^ These beliefs could influence how accurately people can recall and report symptoms in retrospective assessments based on episodic memory. Furthermore, in the study by d’Offay et al.,^30^ participants rated their post-concussion symptoms on a scale from 0 (none) to 4 (severe). Although this scale uses different labels than the RPQ, the scoring range is the same, which may contribute to a stronger association with the RPQ.

The association between the RPQ and the daily symptom scores was similar for the daily registrations of intensity, effect on daily functioning, and effect on QoL. This is somewhat surprising as the RPQ measures the severity of symptoms and not the subsequent effect on daily functioning and QoL. While strong links have been found in youth with PPCS,^32^ in other groups like patients with chronic pain, beliefs about pain can impact QoL more than pain intensity.^33^ Another explanation could be that people might have found it difficult to differentiate between these measures, especially on a day-to-day basis.

Our second aim was to study the variability of PPCS scores over time. We observed substantial fluctuations in PPCS scores over time. For many participants, symptom intensity fluctuated widely, ranging from 0 to 10, within just 3 days. Additionally, the degree of this variability can increase or decrease over time. These findings align with a previous study showing that non-concussed participants displayed substantial individual variability in post-concussion-like symptom scores over time.^34^ The variability in symptom intensity differed greatly between people. For instance, one participant experienced no variability in headache scores, whereas others showed great day-to-day variability in symptom registrations.

These differences in variation were not only observed between participants but also within. For instance, when zooming in on participant two, many of their symptoms varied from zero to seven, while restlessness varied from zero to two. This was also observed on a group level, while fewer people tracked fatigue, the inter-person SD and MSSD were higher, indicating more variability for fatigue than for headache. Fatigue has been found to be a highly variable symptom over time in patients with multiple sclerosis, whereas pain, depressive, and cognitive symptoms also fluctuated, but to a lesser extent.^35^ The individual visual analyses showed that similar RPQ item scores can be related to highly variable daily scores. An RPQ item score of 3 may reflect very different variability in daily symptom scores, for example, daily headache scores might fluctuate more or less than daily fatigue scores within the same individual. When measuring PPCS with the RPQ, all this variability is brought back to one total score, providing an incomplete picture. However, the clinical value of the symptom diary remains to be tested.

Previous research has found that patients with headache, fatigue, or psychosomatic symptoms retrospectively reported symptoms higher than in-the-moment reported symptoms.^36^ We did not find that the highest PPCS scores were more strongly associated with the RPQ than the average score of the daily registrations. However, we did observe some evidence for this in the data, the average intensity score across the 7-day period was 2.20, while for the matched RPQ item scores, this was 2.86. Given the different scoring scales, this suggests that RPQ scores may be approximately twice as high as the daily registrations. In the exploratory visual analyses of the daily registered intensity scores, we also observed that participants who scored a symptom with a four on the RPQ (the highest score) did, for instance, not register daily registrations higher than 6 or 7 (on a scale from 0 to 10). We furthermore saw some evidence of the peak-end effect on an individual basis (the concentration and fatigue scores of participant 11 and the fatigue scores of participant 18).

While the current study aimed to capture the variability of PPCS over time, measuring once a day, this may not fully capture PPCS throughout the day. Multiple daily registrations may, in some instances, provide an even more accurate outcome than one measurement per day.^37^ Future studies could build on this by using higher-frequency sampling to examine within-day fluctuations of PPCS while carefully considering the potential burden on participants. Another point that needs further investigation is the scales that are used. The scales for daily registrations (0–10 numeric rating scale) and the RPQ (0–4 Likert scale) differ, which can influence responses.^38^ While there is no problem in comparing these scales in a correlation analysis, people might interpret such scales differently. In a Likert scale, the options are labeled, while in a numeric rating scale, they can be interpreted based on an individual’s understanding of the numerical range and its meaning. A six might mean something else to two different people. Furthermore, a 0–10 scale naturally allows for more variability in responses. However, the level of variability observed in our data would likely have been apparent even with a 0–4 scale. Future research should investigate which type of scale and labeling is best suited for measuring PPCS symptoms on a daily basis. Lastly, further investigation should also include a comparison with other established PPCS measures, such as the British Columbia Post-Concussion Symptom Inventory.^39^

The findings from the current study suggest that retrospective questionnaires, such as the RPQ, may not adequately reflect the dynamic and fluctuating nature of PPCS. Nonetheless, retrospective measures retain practical advantages, particularly in terms of ease and shorter administration time, making them valuable tools in contexts where time or resources are limited. Assessment approaches such as symptom diary apps, by contrast, may offer a more sensitive method for assessing symptom dynamics, potentially improving both clinical understanding and research accuracy. Rather than viewing these approaches as mutually exclusive, they may be considered complementary tools. Future work should focus on determining the clinical usefulness and contextual relevance of both approaches.

### Strengths and limitations

This is the first study to investigate the relationship between daily and retrospective reporting of PPCS and the variability of PPCS over time. This data provided insights into the heterogeneity of the symptom variability. While participants did not monitor all symptoms included in the RPQ, they selected those most personally relevant. It offers clinical value, as weighting symptom assessments based on personal relevance could lead to more tailored and meaningful symptom monitoring.

This study also has some limitations. First, the mHealth application experienced several technical difficulties, therefore, some data could not be included in the analysis. Although the exact impact is unclear, participants were asked to choose their own symptoms for daily tracking. This makes it unlikely that any symptom would be completely absent, meaning a score of zero for all symptoms on any given day, or a symptom consistently rated as zero throughout the study period (as such cases were excluded). Second, although daily symptom registrations could include reports from up to two days prior, making them partially retrospective, they are nonetheless less reliant on long-term recall than the RPQ. Third, during the 28-day symptom registration period, participants also tested a biofeedback app. As this put an extra burden on participants, this may have affected the adherence rate for symptom reporting. Potential treatment effects or negative side effects could also have affected absolute values of symptom registrations throughout the period, but this was not a focus of the analyses in the current study. Fourth, the current study used the CENTER-TBI version of the RPQ, which asks participants to report symptoms over the past 7 days, whereas most other studies use the 24-h version. This allowed us to compare daily symptom scores of 7 days to the RPQ item scores; however, the results may not be directly comparable to findings from studies using the 24-h recall version. Finally, the sample size of this study consists exclusively of participants with post-traumatic headache and is relatively small, as the sample size was determined for the suitability of feasibility analyses. Future studies with larger sample sizes may enable robust modeling approaches to further disentangle the temporal patterns of PPCS over time.

## Conclusion

The current study shows the potential value of daily symptom diaries as an additional tool for capturing the dynamic nature of PPCS symptoms, offering insights that may be missed by traditional, retrospective questionnaires. PPCS are characterized by symptom fluctuations and great variability, both between and within individuals over time. This variability can be seen in the day-to-day variability of a single symptom of a single person, as well as across different symptoms within the same individual. Trying to capture many highly variable symptoms into one total score is a simplification of a complicated reality. Larger studies are needed to confirm these results.

## Transparency, Rigor, and Reproducibility Summary

The DiSCo study was preregistered (ClinicalTrials ID: NCT05635656). The current secondary analyses were not pre-registered. Data for the present study were collected as part of the DiSCo feasibility study. A total of 23 patients with mTBI were included in the study, and 19 were included in the analyses of the current article. No power calculations were done separately for the present study. The blinding of participants and researchers was not relevant, as this was a daily diary study in which participants completed online registrations and questionnaires. Data acquisition and analyses are reported in more detail in the methods section. All equipment and software used to perform the analysis are widely available (e.g., SPSS and R). No replication or external validation studies have been performed or are planned/ongoing at this time to our knowledge. Deidentified data are available upon reasonable request to the principal investigator at Norwegian University of Science and Technology (AO).

## Copyright Disclaimer

The mHealth app, all data, images, and results are created and owned 100% by NTNU and St. Olavs Hospital.

© 2025 Norwegian University of Science and Technology & St. Olavs Hospital | All Rights Reserved.

## Abbreviations Used

DiSCo: Digital solutions for concussion
ICD-10: International Classification of Diseases
mTBI: mild traumatic brain injury
MMSD: mean squared successive difference
PPCS: persistent post-concussion symptoms
QoL: quality of life
RPQ: Rivermead Post Concussion Symptoms Questionnaire
TRACK TBI: Transforming Research and Clinical Knowledge in Traumatic Brain Injury
